# Convergent genetic adaptation of *Escherichia coli* in minimal media leads to pleiotropic divergence

**DOI:** 10.3389/fmolb.2024.1286824

**Published:** 2024-04-10

**Authors:** Pavithra Venkataraman, Prachitha Nagendra, Neetika Ahlawat, Raman G. Brajesh, Supreet Saini

**Affiliations:** Department of Chemical Engineering, Indian Institute of Technology Bombay, Mumbai, India

**Keywords:** *E. coli*, adaptation, minimal media, RpoB, RpoC

## Abstract

Adaptation in an environment can either be beneficial, neutral or disadvantageous in another. To test the genetic basis of pleiotropic behaviour, we evolved six lines of *E. coli* independently in environments where glucose and galactose were the sole carbon sources, for 300 generations. All six lines in each environment exhibit convergent adaptation in the environment in which they were evolved. However, pleiotropic behaviour was observed in several environmental contexts, including other carbon environments. Genome sequencing reveals that mutations in global regulators *rpoB* and *rpoC* cause this pleiotropy. We report three new alleles of the *rpoB* gene, and one new allele of the *rpoC* gene. The novel *rpoB* alleles confer resistance to Rifampicin, and alter motility. Our results show how single nucleotide changes in the process of adaptation in minimal media can lead to wide-scale pleiotropy, resulting in changes in traits that are not under direct selection.

## Introduction

Populations adapt to an environment by accumulating beneficial genetic changes. The effects of mutations depend on the genetic background (GxG interactions) and this dependency is called epistasis. Due to unknown widespread genetic interactions that lead to epistasis, predicting the effects of mutations, and hence adaptation, is a challenge (Johnson et al., 2023). Despite sequence level dependencies, examples of evolution of populations to show convergence in phenotypes—convergent evolution—have been reported from laboratory experiments (Cunningham et al., 1997; Nakatsu et al., 1998; Cooper et al., 2001; Cooper et al., 2003; Woods et al., 2006; Wong and Kassen, 2011; Herron and Doebeli, 2013; Scribner et al., 2020; Mulvey et al., 2023; Pearl Mizrahi et al., 2023) and nature (Jaekel and Wake, 2007; Chen et al., 2020; Fukushima and Pollock, 2023). Such phenotypic convergence may often be a result of remarkable genetic parallelism, where adaptation is a result of mutations in a specific set of target genes.

Mutational effects also depend on the environment the population is in, due to GxE interactions. When populations well-adapted to an environment are shifted to another, the effects of the mutations they have accumulated may change due to their by-product or pleiotropic effects (MacLean et al., 2004; He and Zhang, 2006; Paulander et al., 2009; Sivakumaran et al., 2011; McGee et al., 2016; Utrilla et al., 2016; Deatherage et al., 2017; Chesmore et al., 2018; Li et al., 2018; Noda-Garcia et al., 2019; Jerison et al., 2020; Kinsler et al., 2020; Ruelens et al., 2023). Such effects can lead to ecological specialization (Schluter, 2009; Forister et al., 2012) and help maintain diversity (Mitchell-Olds et al., 2007; Savolainen et al., 2013). Pleiotropy was thought to operate antagonistically, however, recent ecological evidences show that pleiotropic effects can also be beneficial (Anderson et al., 2011; Remold, 2012; Savolainen et al., 2013; Bono et al., 2017; Elena, 2017). Experiments in laboratory show that while antagonistic pleiotropic effects are possible (Cooper and Lenski, 2000; Turner and Elena, 2000; MacLean et al., 2004; Zhong et al., 2004; Duffy et al., 2006; Bennett and Lenski, 2007; Dettman et al., 2007; Lee et al., 2009; Wenger et al., 2011; Jasmin et al., 2012; Hietpas et al., 2013; Hong and Nielsen, 2013; Jasmin and Zeyl, 2013; Yi and Dean, 2013; Schick et al., 2015; Fraebel et al., 2017), they are not universal (Ostrowski et al., 2005; Bennett and Lenski, 2007; Lee et al., 2009; Jasmin and Zeyl, 2013; Leiby and Marx, 2014; Rodriguez-Verdugo et al., 2014; Li and Zhang, 2018).

Global epistasis provides a framework to predict the effect of a mutation based on the genetic background that it occurs on Diaz-Colunga et al. (2023), and we now know that the effect of a beneficial mutation decreases with increase in the fitness of the background genome it occurs on. However, there exists no such understanding of pleiotropy, rendering its effects unpredictable. Given that environments rarely remain constant or predictable, an understanding of pleiotropy is vital. In this study, we propose to provide an understanding of the link between adaptation and pleiotropy. Specifically, we seek to identify if convergent genetic (and phenotypic) changes lead to predictable pleiotropic effects.

Our experimental setup consists of *Escherichia coli* populations evolving in two environments—glucose and galactose. The utilization of glucose and galactose occur via well-defined routes in *E. coli*. Glucose is broken down into pyruvate via glycolysis. Subsequently, under aerobic conditions, pyruvate enters the TCA cycle, and produce 32 molecules of ATP per molecule of glucose. Galactose, on the other hand, cannot be used for glycolysis directly. Leloir pathway converts it into glucose-6-phosphate, a version convertible into pyruvate molecules. It is then metabolised via glycolysis and TCA cycle (Sellick et al., 2008).

After letting these populations adapt for three hundred generations in their “home” (glucose or galactose) environments, we test the effects of adaptive mutations in each of these populations in “similar” and “dissimilar” non-home environments. Genome sequencing reveals that the genetic basis of adaptation exhibits remarkable convergence, with most lines acquiring mutations in *rpoB* or *rpoC*. Our results show that different alleles of these global regulators fixed in the bacterial populations, leading to statistically identical fitness increases in the evolution environment. Despite conferring this remarkable convergence in adaptive response, the exact pleiotropic effects (in the “away” environment) of the mutations fixed in these populations are unpredictable. Our results show that single nucleotide changes in a global regulator can exhibit divergent pleiotropic effects.

## Methods

### Strain and growth conditions

*Escherichia coli* K-12 (MG1655) strain was used as the parent strain in our experiment. Six independent lines, starting from the ancestor, were started and propagated for 300 generations in minimal M9 media in a specific sugar [0.2% glucose (Sigma Aldrich, Catalogue GA270) or 0.2% galactose (Himedia, Catalogue GRM101)] environment. Cultures were grown to mid-log phase and transferred via a 1:100 dilution to a tube containing 5 mL fresh media. The lines evolved in glucose are referred to as glu1 to glu6; and the lines evolved in galactose are referred to as gal1 to gal6. Minimal media (M9) was used as per the following composition (per litre): Na_2_HPO_4_ (Himedia, Catalogue GRM3961), 6.78g: KH_2_PO_4_ (Himedia, Catalogue TC011), 3g: NaCl (Himedia, Catalogue MB023), 0.5g; NH_4_Cl (Himedia, Catalogue GRM717), 1g; to which filter sterilized 1M MgSO_4_ (Himedia, Catalogue GRM1281) and 0.1M CaCl_2_ (Himedia, Catalogue GRM710) solutions were added.

### Growth rate measurements

Freezer stock of a strain was revived by streaking on an LB plate and allowed to grow overnight at 37°C. A single colony from the plate was then used to inoculate 2 mL LB liquid media, and allowed to grow overnight at 37°C and at 250 rpm. The overnight culture was then sub-cultured 1:100 in M9 media containing the carbon resource of interest at 0.2%, and 150 μL of the culture allowed to grow in a 96-well plate in a microplate (Tecan 1000Pro) reader, with shaking. OD600 readings were taken every 30 min.

### DNA isolation and sequencing

Genomic DNA was isolated by inoculating a single colony of the strain of interest on an LB plate into 2 mL LB and allowed to grow for 6–8 h at 37°C and 250 rpm. Genomic DNA was isolated from the culture as per instructions provided with FavorPrep Tissue Genomic DNA Extraction Mini Kit. Genomic DNA was sequenced by Eurofins Genomics India Pvt. Ltd., Bangalore, on a NextSeq500 platform.

### Rifampicin resistance

Cultures were revived from freezer stock in LB (Himedia, Catalogue M575) and incubated overnight at 37°C and 250rpm. Rifampicin (Himedia, Catalogue CMS1889) stock of 50 mg/mL was made in DMSO. A 1:10 dilution of the cultures was made in LB and different concentrations (200, 100, 50, 25, 12.5, 6.25, 3.125 μg/mL) of Rifampicin were added to it. 150 µL of each of the culture was then transferred in triplicate into a 96-well plate. The minimum inhibitory concentration was checked after 24 h of incubation at 37°C by measuring the optical density.

### *galP* promoter activity assay

The *galP* promoter was cloned upstream of green fluorescent protein (*gfp*) in pPROBE-NT plasmid (Miller et al., 2000). The *galP* promoter sequence was amplified using the primers (5′ AG CGG ATC CCG ATG CTG CCG GTC TGA AGT 3′) and (5′ GG GAA TTC GAT GCC CTC CAA TAT GGT TA 3′) (Miller et al., 2000).

The Δ*galP* knockout was generated using the λ-Red based recombination assay as described in Datsenko and Wanner (2000), using the primers (5′ ATG GCT GAC GCT AAA AAA CAG GGG CGG TCA AAC AAG GCA AGT GTA GGC TGG AGC TGC TTC 3′) and (5′ TTA ATC GTG ACC GCC TAT TTC GCG CAG TTT ACG ACC ATA TGA ATA TCC TCC TTA 3′). The knockout was checked using the primers (5′ CGT GGG AAA AAA CCG ACA AAG C 3′) and (5′ TAC GGT AAG CTG ATG CTC CTG G 3′).

### Mutant *rpoB* allele strains

The mutant *rpoB* alleles in the strains gal1, gal5, and glu5 were transformed into the wild-type ancestor by amplifying the mutant RpoB allele, and replacing the ancestral allele using a λ-Red based recombination. The transformed population was plated on LB plates containing Rifampicin (25 μg/mL), to select for the *rpoB* allele replacement. The PCR product for recombination was amplified using the primers (5′ TTG GCC TGG TAC GTG TAG AGC GTG CGG TGA AAG AGC 3′) and (5′ AAG TTA CCA GGT CTT CTA CGA AGT GGC CTT CTT CAT CCA A 3′).

### Motility plates

Cultures were revived from −80°C freezer stocks in tryptone broth [1.5% tryptone (Amresco, Catalogue J859), 0.5% sodium chloride] and incubated at 37°C, 250 rpm shaking for 12 h. Motility plates [1.5% tryptone, 0.5% sodium chloride, 0.2% agar (SRM, Catalogue 19661)] were poured fresh and 1 µL of the culture was stabbed in the centre of the plate. The plates were incubated at 30°C overnight and imaged using gel doc.

### Statistics

All the error-bars shown in the plots correspond to standard deviations, unless specified otherwise. Y-tests (one or two-tailed) were used to compare means of two data sets and in all cases, the significance level (*p*) was set to 0.05. In cases where multiple hypothesis tests were performed, Bonferroni corrections were performed to avoid false positives.

### Whole-genome sequencing

Genomic DNA of ancestral and all 12 evolution lines was isolated using DNA Mini Kit (Qiagen). Cells from each line were spread on an LB plate for single colonies. A single colony was picked and grown for 6 h in LB at 37°C for DNA isolation. DNA quality and concentration were measured immediately after DNA isolation using Nanodrop Spectrophotometer, and were also confirmed by gel electrophoresis.

All 13 samples (six lines in each environment, and the ancestor) were sent for paired-end sequencing using Illumina NovaSeq 6000, with an average read-depth of 151 bp.

Based on the quality report of fastq files, sequences were trimmed to retain only high-quality sequences for analysis and low-quality sequences were removed. The adapter trimmed reads were aligned to *Escherichia coli* (ATCC 47076) reference genome. Each sample had a minimum coverage of more than ×30. Variant calling was done for the samples using GATK and further annotated using SnpEff. Variants that were present in the ancestral strain were filtered out manually. Raw sequencing data for all 13 samples is available at https://www.ncbi.nlm.nih.gov/sra/PRJNA1022868.

## Results

### Evolution in glucose and galactose showed convergent phenotypic response

The six lines evolved in each of the two environments were tested for growth kinetics in the conditions in which they were evolved at three time points. For glucose-evolved lines, the growth kinetics were characterized after 120, 180, and 300 generations; and for galactose-evolved lines, the growth kinetics were characterized after 60, 180, and 300 generations.

**FIGURE 1:**
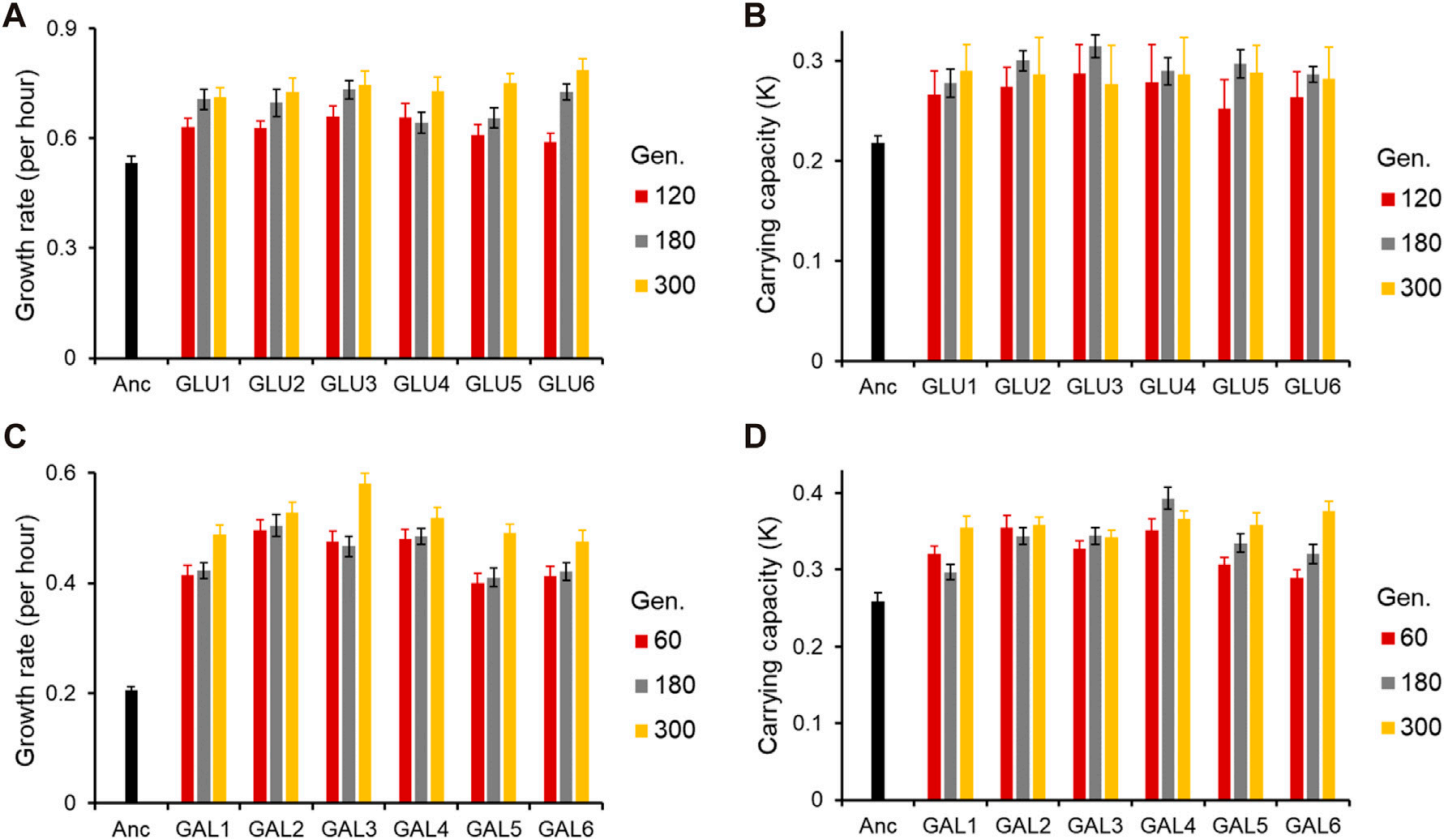
Adaptive response of the evolved lines. **(A)** Growth rate (*r*) and **(B)** Carrying capacity (*K*) of glucose-evolved lines, at 120, 180, and 300 generations, in M9 media containing glucose (home environment). **(C)** Growth rate (*r*) and **(D)** Carrying capacity (*K*) of galactose-evolved lines, at 60, 180, and 300 generations, in M9 media containing galactose (home environment). All experiments were performed three independent times. The average and standard deviation is reported.

As shown in Figure 1 (and Supplementary Figures S1, S2), the adaptive response of the six evolved lines after 300 generations is statistically similar (for all pairwise comparisons of growth rates in the exponential phase, *p > 0.05*).

All six lines evolved in glucose and galactose exhibit exponential phase growth kinetics which is statistically different from the ancestor (*p < 0.05* and *p < 0.01*, respectively). Thus, phenotypic adaptation after 300 generations of evolution, in each of the two environments, was found to be highly convergent.

At 120 generations, all six glucose-evolved lines exhibited a growth rate higher than that of the ancestor (for all pairwise comparisons, *p < 0.05*). Between the six evolved lines, glu6, when compared to lines glu3 and glu4, exhibited a lower growth rate (for both pairwise comparisons, *p < 0.05*). All other pairwise comparisons between the glucose-evolved lines yielded insignificant differences (*p > 0.05*). At 180 and 300 generations, no pairs of evolved lines exhibited significant difference in growth rate (for all pairwise comparisons, *p > 0.05*).

Comparisons of biomass yield in glucose reveals that after 120 generations, no significant differences were observed between any two evolved lines (for all pairwise comparisons, *p > 0.05*). However, all evolved populations exhibited biomass yield which was significantly greater than that of the ancestor (for all pairwise comparisons, *p < 0.05*). After 180 and 300 generations of adaptation, the evolved lines continued to exhibit no difference in biomass yield between themselves (for all pairwise comparisons, *p > 0.05*) and accumulated greater biomass as compared to the ancestor (for all pairwise comparisons, *p < 0.05*).

Similar analysis was also done for the galactose-evolved populations at 60 and 180 generations after adaptation. The galactose-evolved lines at 60 generations exhibit a significantly higher growth rate, when compared against the ancestor (*p < 0.001*). The differences in growth rates between the six lines, however, are statistically insignificant (for all pairwise comparisons, *p > 0.05*). The same pattern was observed at 180 generations, where the growth rate of the evolved lines was statistically significantly higher than that of the ancestor (*p < 0.001*), and the growth rate differences between the evolved lines were statistically insignificant (for all pairwise comparisons, *p > 0.05*).

However, the growth pattern after the exponential phase exhibits a change from 60 to 300 generations. After adaptation for 60 generations, all six lines accumulate biomass which is significantly greater than the ancestor (for all pairwise comparisons, *p < 0.05*). However, unlike growth rate, biomass accumulation exhibits divergence between the evolved lines. Among all pairwise comparisons, only four pairs (gal1 with gal4, gal5 and gal6, and gal3–gal5) were found to have similar biomass yield (for all four pairwise comparisons, *p > 0.05*). All other pairwise comparisons between the evolved lines yield *p < 0.05*. After 300 generations of adaptation, however, only line gal3 exhibits a different biomass yield (as compared to any of the other five evolved lines, *p < 0.05*). All other lines exhibit accumulate biomass similarly (for all pairwise comparisons, *p > 0.05*).

### Fitness trends in non-home environments with alternative carbon sources are not identical

**FIGURE 2:**
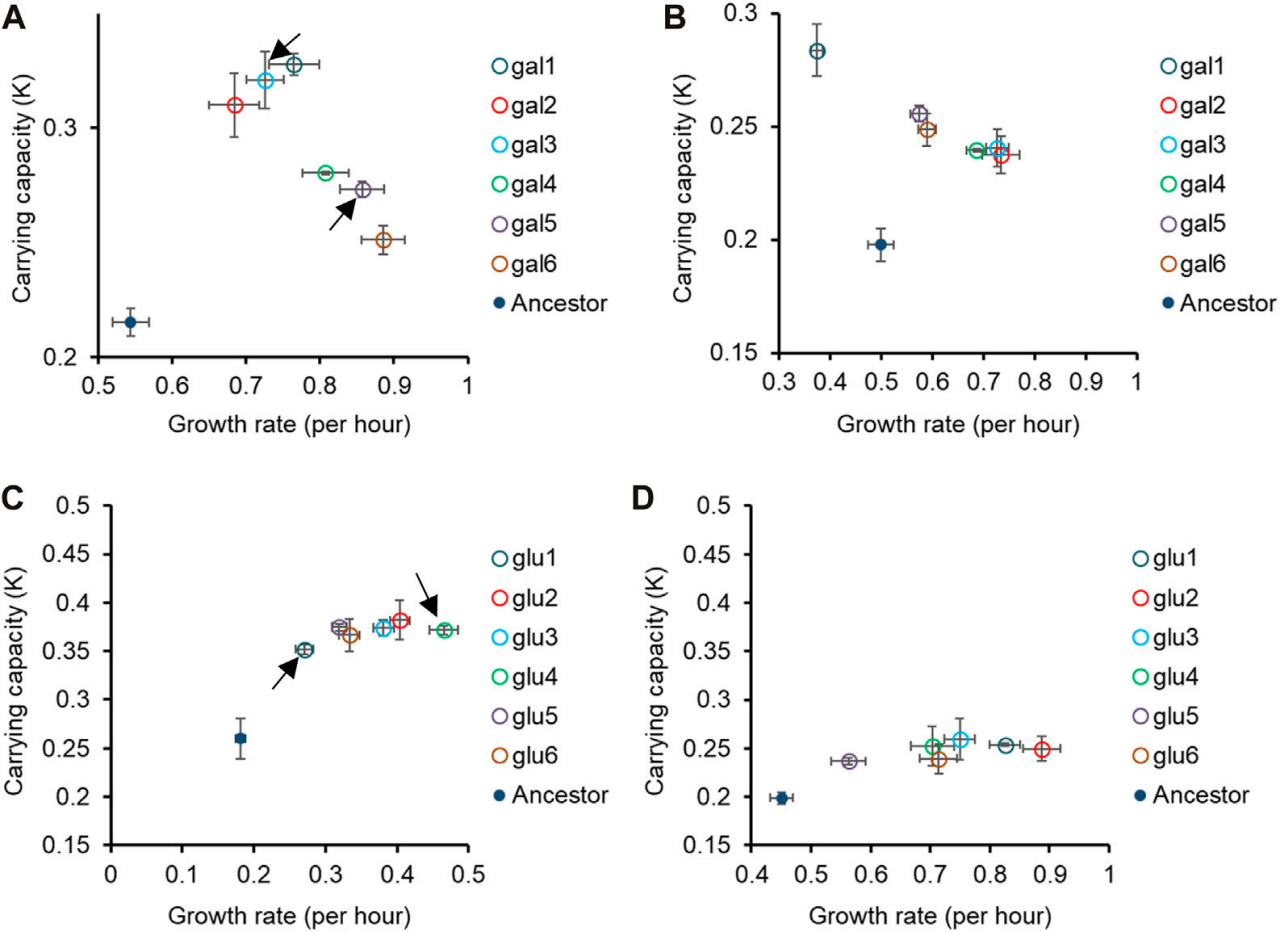
Pleiotropic effects of adaptation in home carbon environments. Growth rate (*r*) and carrying capacity (*K*) of glucose-evolved lines at 300 generations, in M9 media containing **(A)** galactose and **(B)** lactose. Growth rate (*r*) and carrying capacity (*K*) of glucose-evolved lines at 300 generations, in M9 media containing **(C)** galactose and **(D)** lactose. In **(A,C)**, arrows indicate the two glucose- and galactose-evolved lines selected for further analysis. All experiments were performed three independent times. The average and standard deviation is reported.

**FIGURE 3:**
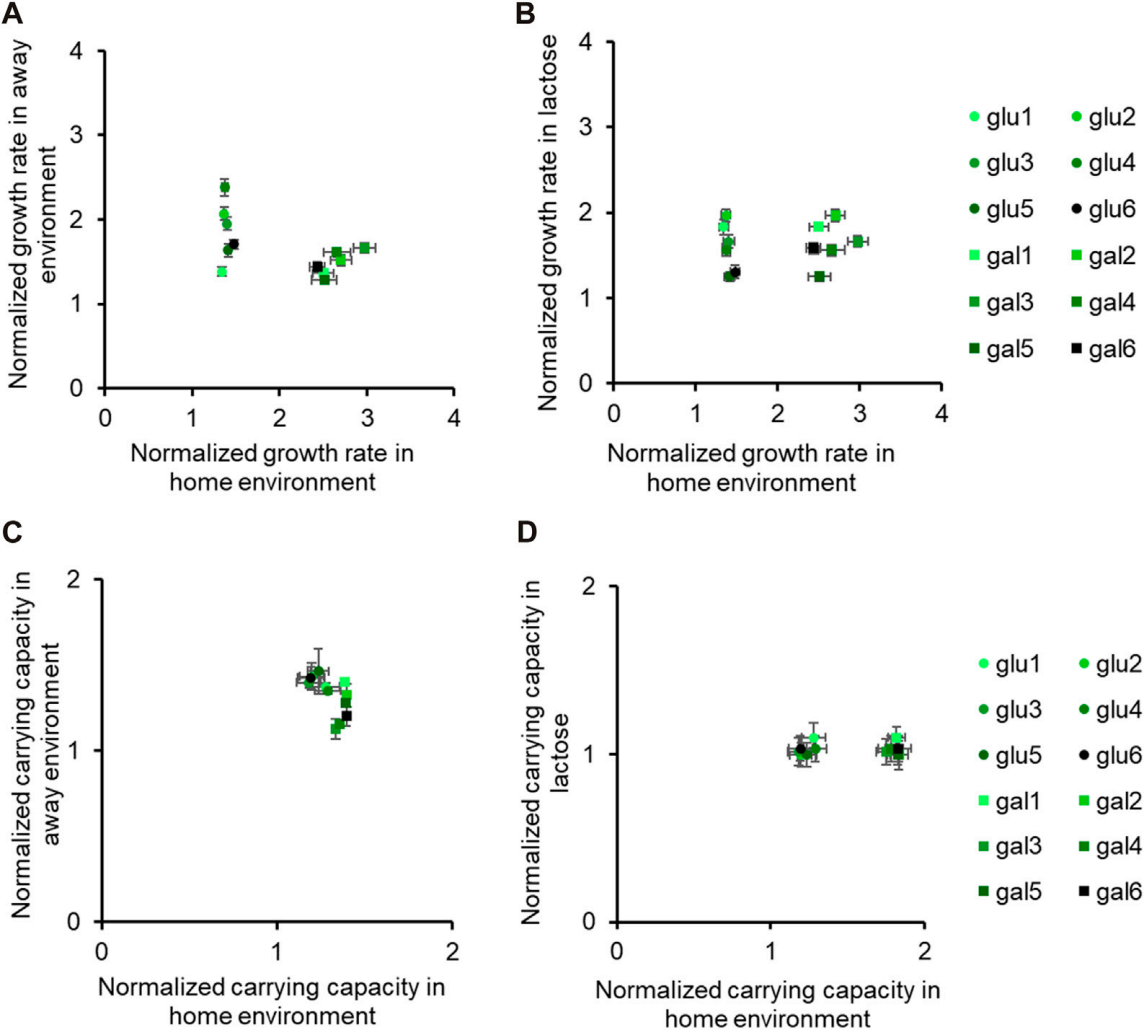
Fitness effects in “similar” non-home environments are not predictable based on fitness in home environment. All six glucose-evolved and galactose-evolved populations were tested for growth in M9 minimal media containing alternative sources of carbon. Growth rates and carrying capacities were normalized with that of the ancestor in the environment tested. Away environment for glucose-evolved lines is galactose, and that for galactose-evolved lines is glucose. **(A,B)** are used to compare the normalized growth rates of the twelve populations in their home environment with that in the away environments. Similarly, **(C,D)** show how the normalized carrying capacity changes in different environments.

In Figures 1, 2, we demonstrate that the glucose- and galactose-adapted populations exhibit convergent growth dynamics, when grown in their respective environments, galactose, and glucose. We next tested for growth of the glucose-evolved and galactose-evolved populations in an away environment, that differed in the source of carbon (glucose, galactose, or lactose) and report the results in Figures 2, 3 and Supplementary Figure S3.

While all the evolved populations had accumulated mutations that were beneficial in these environments as well, their exact fitnesses were not predictable based on growth in the home environment.

We compared the growth rates of these evolved populations and found that the lines gal1 and gal5 had dissimilar growth rates in glucose (*p < 0.001*), but identical growth rates in lactose (*p > 0.05*). This observation presents as evidence of pleiotropic effects of adaptation.

Similar effects of pleiotropy were seen in glucose-evolved lines, when grown in galactose and lactose. The growth rate of lines glu4 and glu5, when grown in galactose, was statistically significantly different from each other (*p < 0.001*). On the other hand, the growth rate and optical density of the same two lines, when grown in lactose, was statistically identical (*p > 0.05*).

Since the above-discussed two pairs of lines—gal1 and gal5 and glu4 and glu5—exhibit the most diverse phenotypes when tested in glucose and galactose, respectively, we analyse the pleiotropic effects of adaptation in glucose and galactose in these lines in two other contexts. Before explaining the effects of pleiotropy in these four lines, we first identified the genetic basis of adaptation in these twelve evolution lines.

### Genome sequencing

To test the genetic basis of the adaptive response described above, we sequenced the genomes of all twelve evolved lines, and compared it with the ancestor. The list of the mutations in the evolved lines are as shown in Table 1. The sequencing results demonstrate that, as has been previously observed (Conrad et al., 2010a; LaCroix et al., 2015; Wannier et al., 2018; Bakerlee et al., 2021), *rpoB* and *rpoC* are major mutational targets of adaptation in minimal media. For glucose-adapted lines, four out of the six lines acquired mutation in either *rpoB* or *rpoC*. On the other hand, out of the six galactose-adapted lines, five acquired mutation in either *rpoB* or *rpoC.*

**TABLE 1:** List of mutations in the evolved lines. **(**Genomic positions are indicated in brackets).

Line number	Glucose-evolved	Galactose-evolved
1	***rpoB* [**H526Y (C1576T)**]** (4339879)	***sspA*** [L83R (A247C)] (506312)
		***rrfF*** [T109G] (Position: 3421456)
		***rrfD*** [C4A] (Position: 3421806)
		***rpoB*** [S522Y (C1565A)] (4339883)
2	***kgtP*** [upstream variant −431 C to A] (1157554)	***nuoF*** [T993G (H331G)] (1483168)
3	***nanR*** [upstream variant −706 C to T] (4580665)	***rpoB*** [G2137A (G713S)] (4339318)
4	***rpoC* [**D622E (C1866G)**]** (4335484)	***rpoC*** [C3418T (R1140C)] (4333932)
5	***rpoB* [**H526Y (C1576T)**]** (4339879)	***rpoB*** [N613H (A1838C)] (4339818)
		***galP*** [C→A (82 bases upstream of start codon)] (795467)
6	***rpoB* [**H526Y (C1576T)**]** (4339879**)**	***rpoC*** [C2932T (R978C)] (4334418)

Mutations in *rpoB* and *rpoC* have previously been isolated, during adaptation in minimal media (Conrad et al., 2010a; LaCroix et al., 2015). In a single experiment, as many as 37 out of 45 lines adapted in minimal media acquired a mutation in *rpoB* or *rpoC* (Conrad et al., 2010a). Interestingly, these mutations include not just SNPs, but also deletions of specific regions of *rpoC* (Herring et al., 2006).

As discussed above, we focus on two lines evolved in glucose (glu4 and glu5) and two lines evolved in galactose (gal1 and gal5). The choice of these lines for sequencing was dictated by the observation that their growth kinetics in the foreign environment was most different from each other’s (as indicated in Figure 2 and Supplementary Figure S3).

The glucose-adapted lines have mutation in only a single gene: *rpoC* in glu4 line and *rpoB* in glu5 line. Both the lines carry one non-synonymous mutation each. The galactose-evolved lines (gal1 and gal5), on the other hand, had more than one mutation each. Each of the two galactose-evolved line, however, also had a non-synonymous mutation in *rpoB*. Thus, three of the four evolved lines analysed further in this work have a mutation in *rpoB*. Mutations in RNA Polymerase (RNAP) components have been demonstrated to be adaptive under nutrient, thermal, radiation, and antibiotic stresses (Severinov et al., 1993; Herring et al., 2006; Conrad et al., 2010b; Tenaillon et al., 2012; Degen et al., 2014; Harden et al., 2015; Bruckbauer et al., 2019; Lennen et al., 2023).

In addition to mutations in *rpoB*, the galactose-evolved lines gal1 and gal5 also have mutations in *sspA* (related to starvation response), *galP* promoter region, and rRNA genes. Genes associated with these mutations are known to be associated with cellular response to growth in stressful conditions (Abo et al., 2002; Christensen and Gerdes, 2003; Moore and Sauer, 2007; Ranquet and Gottesman, 2007; Ono et al., 2009; Li et al., 2013).

To test the role of the *rpoB* alleles in dictating aspects of cellular physiology, we shifted the *rpoB* alleles to the ancestral background. The *rpoB* mutants isolated from adaptation in minimal media are known to confer antibiotic resistance to Rifampicin (Alifano et al., 2015; Li MC. et al., 2021). Hence, the *rpoB* alleles were amplified and transformed in an ancestor carrying the plasmid pKD46 (Datsenko and Wanner, 2000). The transformed cells were selected on Rifampicin plates, which contained 40 μg/mL of the antibiotic. All three *rpoB* alleles identified in this study were transferred to the ancestor genetic background in this fashion, and the constructs were verified via sequencing. The pleiotropic effects of the mutant alleles were characterized as described below.

### *RpoB* alleles confer rifampicin resistance to different extents

Many *rpoB* alleles have been previously demonstrated to alter the sensitivity of the cell to the antibiotic, rifampicin (Ezekiel and Hutchins, 1968). The minimum concentration of Rifampicin which permitted growth was established as the MIC associated with each of the three isolated *rpoB* allele. As shown in Table 2, the MIC associated with all three *rpoB* alleles reported in this study is greater than of the ancestral *rpoB* allele.

**TABLE 2:** Rifampicin MIC for different *rpoB* alleles.

*rpoB* allele	Rifampicin MIC
Ancestor	25 μg/mL
H526Y (C1576T)	>200 μg/mL
S522Y (C1565A)	>200 μg/mL
N613H (A1838C)	50 μg/mL

Mutations in *rpoB* which confer resistance to Rifampicin are classified into four groups—N terminal cluster (143–148), cluster I (505–537), cluster II (562–575) and cluster III (684–690) (Campbell et al., 2001). Two of the three *rpoB* alleles we report have single amino acid changes in cluster I. One of the mutants (H526Y) has been reported before (Lisitsyn et al., 1984) and is said to alter the rifampicin binding pocket in *Mycobacterium tuberculosis* (Molodtsov et al., 2017). The crystal structure of Rif-RNAP reveals that rifampicin touches closely the base of a loop which is flanked by the cluster I amino acids (Campbell et al., 2001). As a result, due to steric hindrance caused by mutations in this region, rifampicin fails to bind to RNAP, yielding the cell insensitive to the antibiotic. The third mutation (N613H) does not lie in any of these clusters, and little is known about how such a “distant” change can confer resistance.

### *RpoB* alleles cause change in flagellar gene expression and motility

It has been suggested previously that the increase in fitness in minimal media can be due to a reduction/loss in motility (Fong et al., 2005; Leatham et al., 2005). Since *rpoB* alleles confer changes to cellular physiology and gene expression in a global sense (Conrad et al., 2010a), we tested how the three *rpoB* alleles change the ability of *E. coli* to swim in liquid media. Alleles of *rpoB* have previously been shown to lead to a decrease in the gene expression of genes associated with flagellar biosynthesis, and as a result, reduced motility on plates (Conrad et al., 2010a; Meenakshi and Munavar, 2018).

**FIGURE 4:**
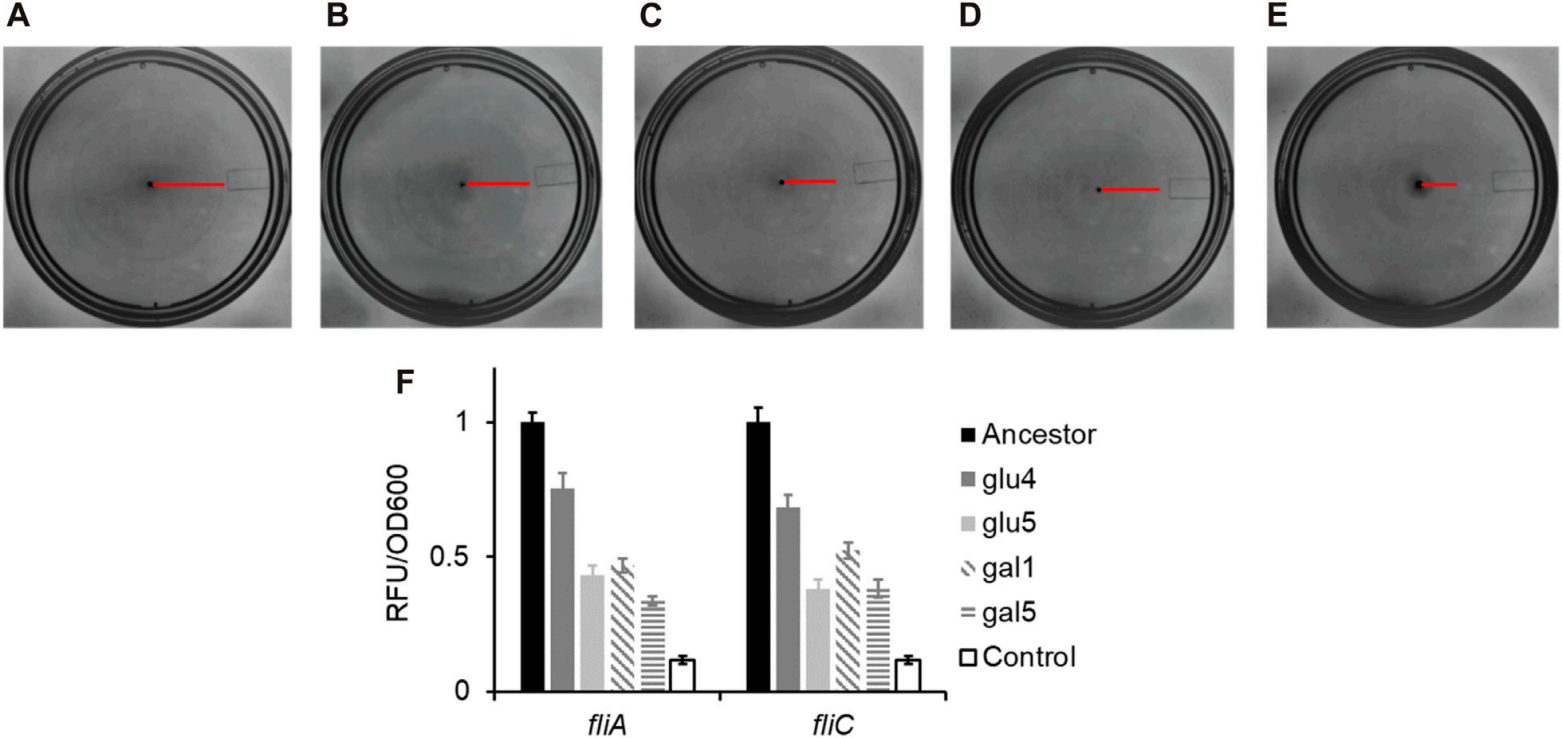
RpoB alleles reduce the ability of *Escherichia coli* to swim in liquid media, and lower gene expression from the flagellar promoters *fliA* and *fliC*. **(A–E)** Motility rings of ancestor **(A)**, GLU4 **(B)**, GLU5 **(C)**, GAL1 **(D)**, and GAL5 **(E)** after 8 h of growth on Tryptone Broth motility plates at 30°C. **(F)** Expression from the P*fliA* and P*fliC* promoters in the strains with ancestral and the mutant *rpoB* alleles. All experiments were performed in triplicate. The average values and standard deviation are reported.

As shown in Figures 4A–E, the swimming ability of the strains carrying any of the three *rpoB* alleles identified in this study was compromised, as compared to the ancestral strain. Flagellar biosynthesis and the expression of the chemotaxis proteins are under the control of a single transcriptional regulatory complex, FlhD_4_C_2_ (Neidhardt, 1996). *Escherichia coli* “decides” to be motile or not, based on integration of environmental and cellular signals at the *flhDC* promoter and transcript (Bertin et al., 1994; Shin and Park, 1995; Soutourina et al., 1999; Ko and Park, 2000; Lehnen et al., 2002; Sperandio et al., 2002; Francez-Charlot et al., 2003). FlhD_4_C_2_ activates the expression of the flagella-specific sigma factor, FliA. FliA, in turn, activates its own expression and that of several genes, including *fliC*, which encodes for the flagellar filament (Chilcott and Hughes, 2000). The promoter activity in the strains carrying the three mutant *rpoB* alleles, the P_*fliA*_ and P_*fliC*_ promoter activity was reduced as compared to the ancestor (Figure 4F). All four evolved strains exhibit statistically significantly lower class 2 and class 3 gene expression, compared to the ancestor (*p < 0.01* for glu4, *p < 0.001* for glu5, *p < 0.001* for gal1, and *p < 0.001* for gal5).

Consistent with the observations reported in the past, the alleles that fixed in our evolving populations also exhibit decreased motility, via a downregulation of the class 2/3 promoter (P*fliA*) and a class 3 promoter (P*fliC*) in the flagellar cascade.

### *galP* promoter mutation increase transcriptional activity from the promoter

One of the mutations in the gal5 line was in the promoter region of the gene *galP* (Table 1). *GalP* is responsible for transport of galactose from the extracellular environment into the cell (Macpherson et al., 1983). From the perspective of adaptation in galactose, an increased expression of the galactose transporter should lead to an increase in fitness. To test whether the mutation in the *galP* promoter increases rate of transcription, we fused the ancestral and the evolved *galP* promoter with *gfp* and tested for fluorescence levels in the presence of galactose.

**FIGURE 5:**
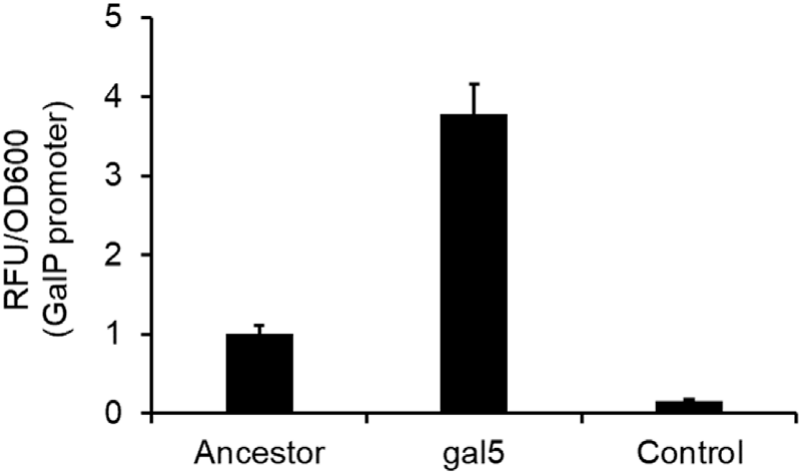
P*galP* promoter activity is increased by the mutation in the promoter region. *galP* promoter activity, as measured by the promoter fusion with *gfp*, shows that the promoter mutation increases expression from the promoter ∼4-fold, as compared to the ancestral *galP* promoter (*p < 0.01*). The experiment was performed in triplicate. The average value and standard deviation are reported.

Our results showed that the mutated *galP* promoter exhibits a significantly higher level of fluorescence, as compared to the ancestral *galP* promoter (Figure 5). Thus, a part of increase in fitness of the galactose-evolved lines in galactose environment is contributed to by the increased levels of the *galP* protein.

## Discussion

Populations adapt to an environment under the action of evolutionary forces like mutations, natural selection, and genetic drift. While natural selection acts to increase the frequency of beneficial mutations, drift randomly alters the frequency of alleles. In such a context, it is the most beneficial alleles that survive drift. Despite the role of chance events in dictating the trajectory of evolving populations (Lee et al., 2009; Jasmin and Zeyl, 2013; Bono et al., 2017), adaptive convergence, at a phenotypic and genotypic level, has been observed in several microbial evolution experiments (Cunningham et al., 1997; Nakatsu et al., 1998; Cooper et al., 2001; Cooper et al., 2003; Wong and Kassen, 2011; Herron and Doebeli, 2013; Scribner et al., 2020; Lambros et al., 2021; Mulvey et al., 2023; Pearl Mizrahi et al., 2023; Tjendra et al., 2023). Such experimental evidences show that evolution in certain conditions, given a genetic background and environment, could be predictable, even at the genetic level.

In the absence of information about genetic background and environment, it becomes impossible to predict the exact effect of a mutation because of epistasis (GxG) and pleiotropy (GxE). Genetic interactions (GxG) are pervasive, and lead to widespread epistasis across the genome (Diaz-Colunga et al., 2023). These epistatic relations also change depending on the environment, leading to another layer of complexity in the form of GxGxE interactions. Using theoretical and empirical approaches, we now know from global epistasis patterns that despite sequence-level unpredictability, beneficial effects of a mutation decrease with an increase in background fitness (Wiser et al., 2013; Kryazhimskiy et al., 2014). Although a mechanistic explanation to such an observation does not exist, a statistical framework based on the distribution of fitness effects enables prediction of evolutionary trajectories (Sarah et al., 2023). However, no such model exists to understand GxE interactions, or pleiotropic effects of mutations. Several experimental studies have reported pleiotropy in a wide-range of cases (Chen et al., 2020; Jerison et al., 2020; Bakerlee et al., 2021). Recent laboratory evolution-based studies to understand pleiotropy have commented on the role of initial fitness, ploidy, and environment (Jerison et al., 2017; Bakerlee et al., 2021; Chen et al., 2023). While it is agreed that several by-product effects of mutations are deleterious, antagonism is not universal, and the prediction of pleiotropy remains a challenge. We ask if adaptive trajectories in a particular environment can help predict pleiotropy, in “similar” or “dissimilar” non-home environments.

In an attempt to identify if pleiotropic effects can be predicted based on adaptation, we evolve *E. coli* in an environment consisting of one sugar source (0.2% glucose or galactose) in M9 minimal media, for a period of 300 generations. By using a simple organism such as *E. coli*, we eliminate the complexities that come with eukaryotic model organisms. We know from previous works that evolution of *E. coli* in stressful conditions leads to genetic changes in global regulators (Rudolph et al., 2010; Weinstein and Zaman, 2019; Li F. et al., 2021). Specifically, different alleles of global regulators like *rpoB* and *rpoC* are shown to be adaptive in environments where temperature is elevated, or an antibiotic is present (Rudolph et al., 2010; Weinstein and Zaman, 2019). We investigate whether the mutational targets in our evolving populations were nutrient utilization genes, or global regulators, or both. After evolving for 300 generations, nine out of these twelve populations accumulated mutations in the *rpo* genes. In fact, seven of these nine populations did not have any other mutations. Other mutational targets included genes involved in starvation response, ribosome synthesis, and nutrient uptake, as expected.

Despite the sequence level differences, cells evolving in an environment showed extremely similar speeds of adaptation, and fitness change at the end of 300 generations. This was a classic case of convergent evolution, both at phenotypic and genotypic levels.

Since adaptive mutations occurred in global regulators, we checked if the fitness of the evolved populations, in two types of environments (non-home environments), was identical to that in the home environment. First, we tested how changing the sugar source in the environment could alter fitness. This constituted our test of pleiotropy in a highly “similar” environment. Given that the evolution of these populations occurred in an environment where selection acted on nutrient utilization, we expected insignificant changes in the fitness of these populations when shifted to environments with other sugars.

Growth assays showed that no mutation that fixed in these evolving populations was deleterious in the away environments. In our experiments, some evolved cells showed diverse responses in non-home environments as observed in literature (Travisano et al., 1995)—two glucose-evolved lines (glu4 and glu5) grew similarly in lactose but differently in galactose, and two galactose-evolved lines (gal1 and gal5) grew similarly in lactose but differently in glucose. Three of these populations had one SNP each in *rpoB*, while the fourth one had a SNP in a *rpoC*. Despite all these populations having adapted identically in minimal nutrient media, they do not show identical pleiotropic effects in environments containing different sugar sources. Therefore, our results show that extending the implications of adaptation in one sugar environment to another should be done with caution.

Second, we probed the fitness of these evolved populations in environments that posed a non-nutrient stress, i.e., in highly “dissimilar” environments. Since *rpoB* mutations have been reported to confer resistance to Rifampicin, we quantified the minimum inhibitory concentration of the evolved glu4, gal1, and gal5 populations. Again, the fitness of these evolved cells in the presence of Rifampicin was not predictable based on the fitness in the home environment—all three populations had different minimum inhibitory concentrations of the antibiotic. We report three new alleles of the *rpoB* gene, and show that quantitative pleiotropic effects of adaptation are not gene-dependent, but change with mutations at the nucleotide level. Therefore, an attempt to quantify pleiotropy in a high-throughput fashion using deletion collections, like done in the past (Dudley et al., 2005), could yield highly inaccurate predictions of quantitative fitness effects in away environments.

Mutations in global regulators facilitate adaptation by altering gene regulation patterns (Fong et al., 2005; Saxer et al., 2014; Rodriguez-Verdugo et al., 2016; Gonzalez-Gonzalez et al., 2017; Iyer et al., 2021; Mahilkar et al., 2021). Altered motility is one of the outcomes of change in gene regulation (Conrad et al., 2010a; Perez-Varela et al., 2017; Cutugno et al., 2020). None of the populations which harboured a mutation in the *rpoB* gene showed similar motility.

Pleiotropic effects are known to slow down or speed up adaptation, depending on the precise relation between the genotype and selection acting in the environment (Hamala et al., 2020). Empirical investigations to ascertain the nature of pleiotropic effects of adaptation show that pleiotropy could be both deterministic or idiosyncratic (Jerison et al., 2020; Bakerlee et al., 2021). We have tested pleiotropic effects of evolution in a simple environment in different environments—one in which a nutrient source was changed, and another in which an antibiotic was present. Overall, in the two contexts, pleiotropic effects were only beneficial, making possible the prediction of the qualitative nature of pleiotropic effect. However, the exact by-product fitness effects, even of single mutations in global regulators, were not predictable based on adaptation (in four out of twelve populations) that resulted in identical mutational targets. In the small set of environments that we tested, we also observed that pleiotropy emerges early, as reported in Bakerlee et al. (2021), and that the generalist or specialist behaviour of a population depends on the pair of environments in which pleiotropy is being investigated, even if the pairs imposed “similar” selection pressures on the population. Given that unpredictability is not ruled out, our results highlight that in order to build a statistical understanding of pleiotropic effects, sequence-level mapping of mutations with several environmental pairs, of various types, is necessary.

## Data Availability

The raw data supporting the conclusion of this article will be made available by the authors, without undue reservation.
