# Supplementary material for: Convergent genetic adaptation of Escherichia coli in minimal media leads to pleiotropic divergence
Source: Front Mol Biosci. 2024 Apr 10;11:1286824. doi: 10.3389/fmolb.2024.1286824 (PMC11039892; doi:10.3389/fmolb.2024.1286824)
Supplement: Supplementary file 1 [file Image1.pdf]

Supplement: Convergent genetic adaptation of *Escherichia coli* in minimal media leads to pleiotropic divergence.

Pavithra Venkataraman[1], Prachitha Nagendra[1], Neetika Ahlawat[1], Raman G. Brajesh[1,2], and Supreet Saini[1,]*

[1] Department of Chemical Engineering, Indian Institute of Technology Bombay, India 400 076
[2] Current Address: Department of Biomedical Engineering and Bioinformatics, Chhatisgarh Swami Vivekanand Technical University, Newai, Durg 491 107

A.

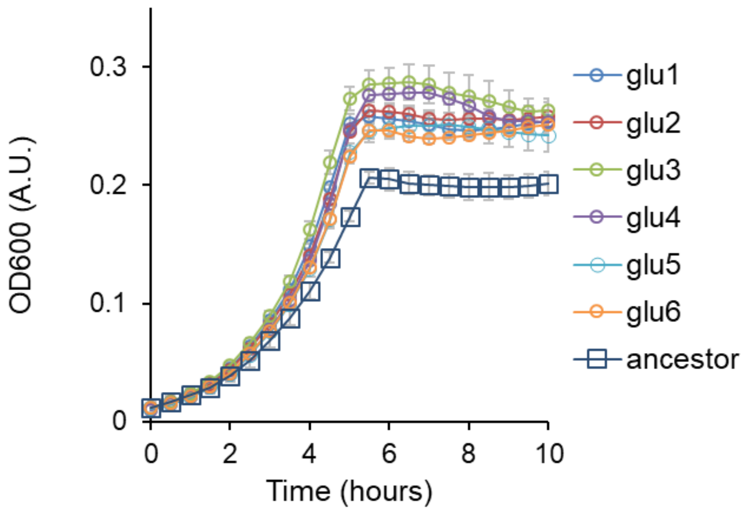

B.

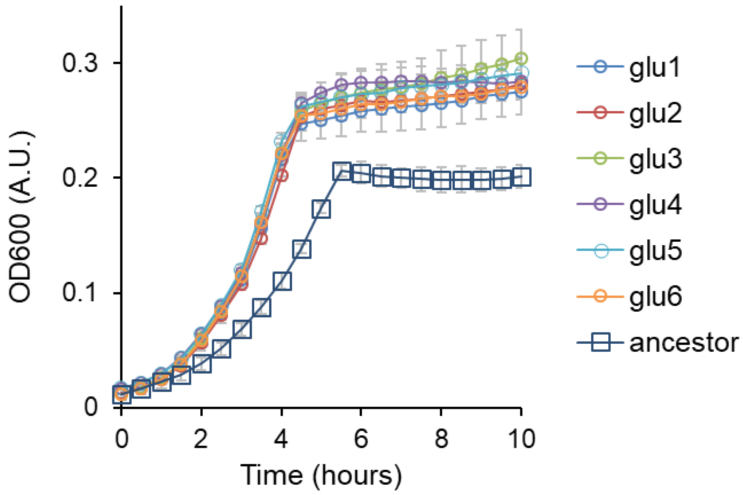

C.

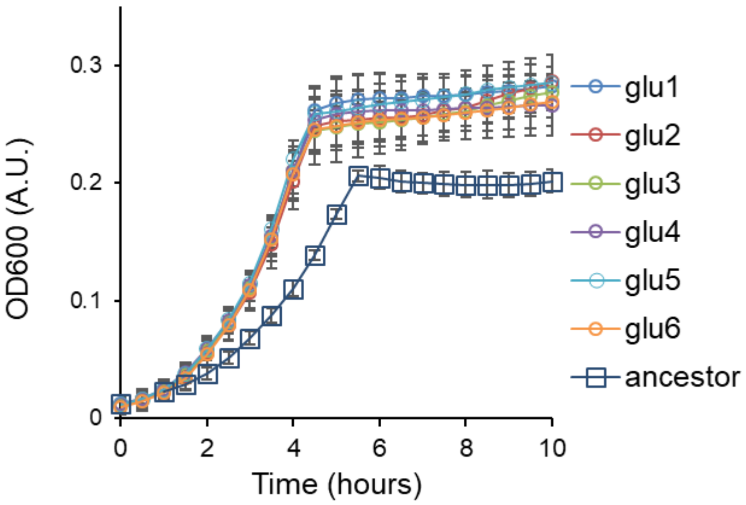

Figure S1. Growth kinetics of the glucose-evolved lines in '*home*' environment after **(A)** 120, **(B)** 180, and **(C)** 300 generations of adaptation. All experiments were performed three independent times. The average and standard deviation is reported.

A.

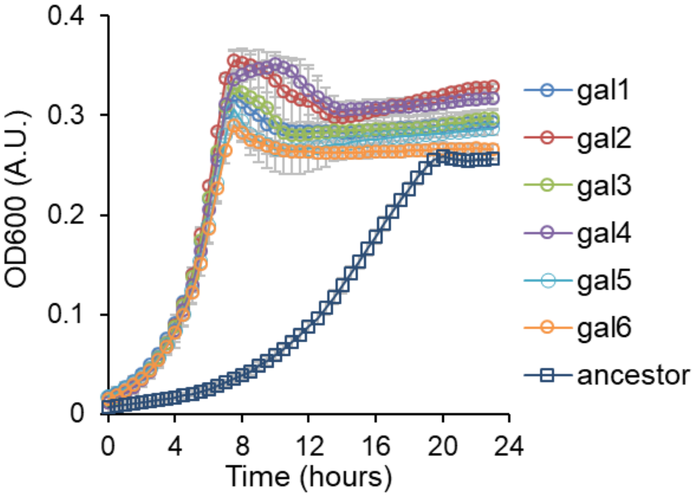

B.

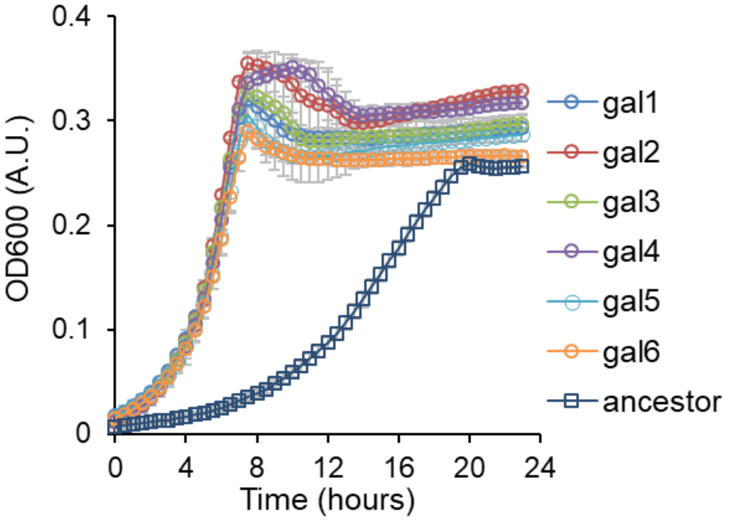

C.

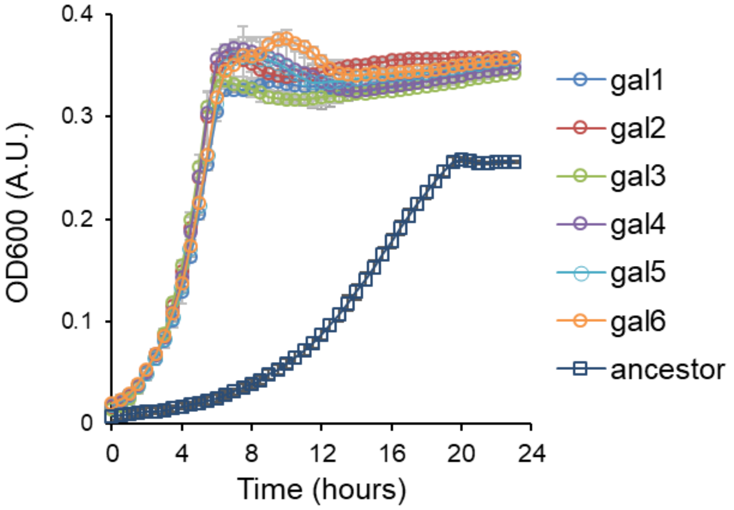

Figure S2. Growth kinetics of the galactose-evolved lines in '*home*' environment after **(A)** 60, **(B)** 180, and **(C)** 300 generations of adaptation. All experiments were performed three independent times. The average and standard deviation is reported.

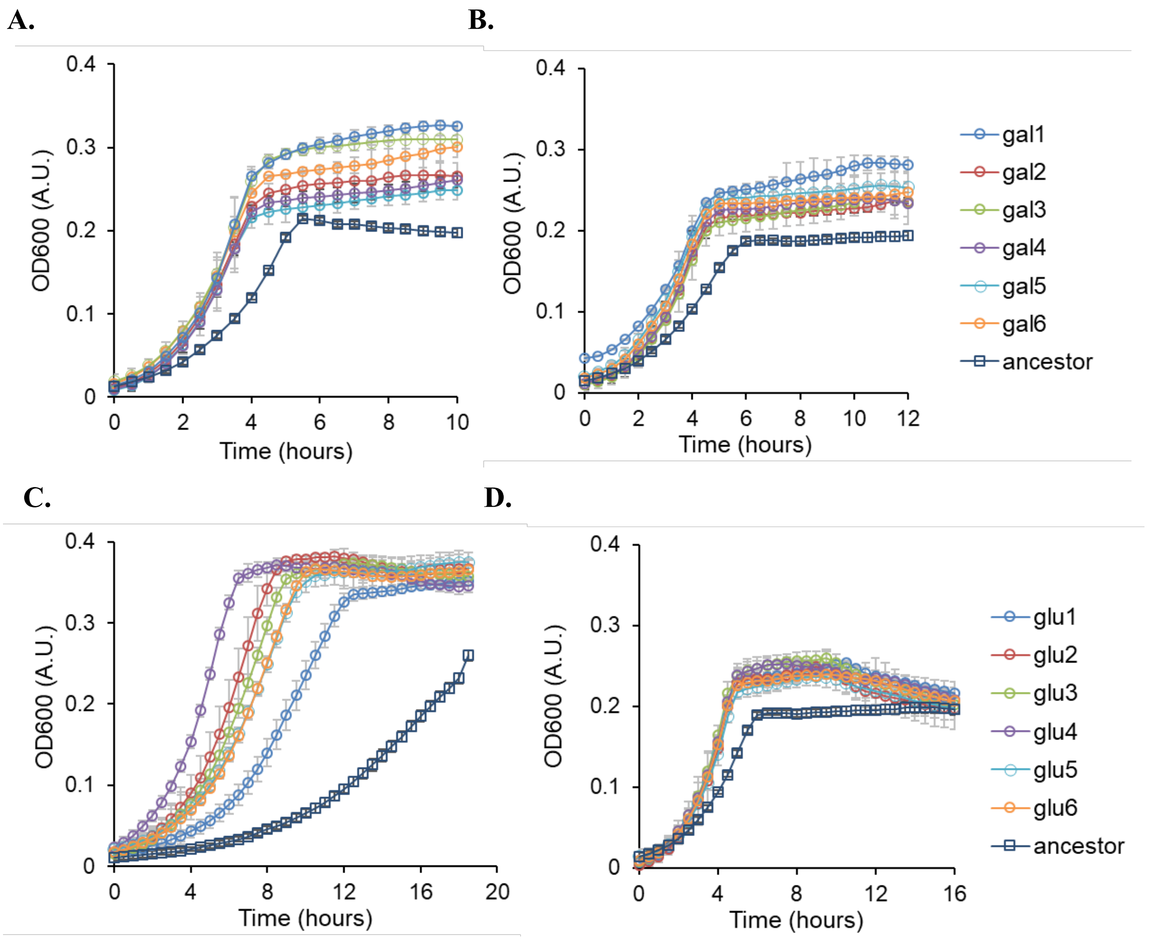

Figure S3. Growth kinetics of evolved lines in alternate carbon source environments may exhibit a distinct growth dynamic. Growth kinetics of galactose-evolved lines in M9 media containing **(A)** glucose and **(B)** lactose as the carbon source, respectively. Growth kinetics of glucose-evolved lines in M9 media containing **(C)** galactose and **(D)** lactose as the carbon source, respectively. Squares represent the ancestor, and circles represent the evolved lines. All experiments were performed in triplicate. The average and standard deviation are reported.
